# ﻿A description of a new species of *Mongolodiaptomus* Kiefer, 1937 (Copepoda, Calanoida, Diaptomidae) from Thailand with an up-to-date key to the genus

**DOI:** 10.3897/zookeys.1209.125838

**Published:** 2024-08-06

**Authors:** Laorsri Sanoamuang, Kamonwan Koompoot

**Affiliations:** 1 Laboratory of Biodiversity and Environmental Management, International College, Khon Kaen University, Khon Kaen 40002, Thailand Khon Kaen University Khon Kaen Thailand; 2 Applied Taxonomic Research Center, Faculty of Science, Khon Kaen University, Khon Kaen 40002, Thailand Khon Kaen University Khon Kaen Thailand

**Keywords:** Aquatic habitats, biodiversity, distribution, endemic, *Mongolodiaptomusphutakaensis*, Southeast Asia, taxonomy

## Abstract

The genus *Mongolodiaptomus* is widely distributed in stagnant water bodies in Southeast Asia. During a comprehensive collection of freshwater copepods from different areas in Thailand, a previously unknown species of calanoid copepod, *Mongolodiaptomusphutakaensis***sp. nov.**, was recorded. Representatives were found in a natural swamp located in the Kok Phutaka community forest in Khon Kaen Province, northeastern Thailand. The new species belongs to the “*M.loeiensis* species group” and most closely resembles *M.loeiensis* and *M.mekongensis* by having a distinct shape of the second exopodal segment of the male right P5, with enlarged proximal and distal parts of the outer margin as well as a bent and twisted principal lateral spine. The new species can be distinguished from its congeners by various characters in the males. The ventral surface of the right caudal ramus has two chitinous teeth and two knobs. The intercoxal plate is slightly produced distally and without any spine. The right P5 basis lacks a hyaline membrane on the inner margin but has a distinct spur-like chitinous process at the mid-distal margin on the posterior surface. The left P5 basis has a thin, longer hyaline lamella on the inner margin. The new species is rare, having been observed in only one out of approximately 5,000 surveyed locations in Thailand. A detailed morphological comparison and an up-to-date key to the *Mongolodiaptomus* species are presented. Their taxonomic characters, interspecies relationships, and biogeography are discussed.

## ﻿Introduction

In 1937, Kiefer created the genus *Mongolodiaptomus* to include a group of Asian freshwater diaptomid copepods, with *Mongolodiaptomusformosanus* Kiefer, 1937 as the type taxon (Kiefer 1937; Walter and Boxshall 2024). Ranga Reddy et al. (2000) recommended to use the ornamentation of the right second exopod of the male P5 as an important character to distinguish between diaptomid copepods, especially those from the three closely related genera *Neodiaptomus* Kiefer, 1932, *Allodiaptomus* Kiefer, 1936, and *Mongolodiaptomus* Kiefer, 1937. Thus, the characters used to distinguish the males of *Mongolodiaptomus* from related genera is the presence of at least two lateral spines on the second exopod of the right P5, one principal spine in the middle of the segment, and one or two accessory spines proximally or distally on the outer margin.

At present, the genus *Mongolodiaptomus* consists of 11 valid species, distributed across Asia, with countries in the lower Mekong River Basin as the epicenter (Sanoamuang and Watiroyram 2018). However, this is not the final number, since there are two more species with doubtful identities. Ranga Reddy et al. (1998) observed some morphological variabilities in *M.botulifer* (Kiefer, 1974) from Thailand, leading to serious doubt about the validity of the closely allied *M.malaindosinensis* (Lai & Fernando, 1978). Despite the similarities in morphological features, Sanoamuang and Dabseepai (2021: 20) provide detailed explanations that distinguish the two congeners from each other. The validity was confirmed in the previously mentioned paper. Ranga Reddy et al. (1998) originally described *M.rarus* (Ranga Reddy, Sanoamuang & Dumont, 1998) as *Allodiaptomusrarus*, based on a single male specimen from Thailand. This species was later transferred to the genus *Mongolodiaptomus* by Sanoamuang (2001). Since there are no type specimens of *M.malaindosinensis* available and no description of the female morphology of *M.rarus*, it would be advisable to redescribe *M.malaindosinensis* and *M.rarus*, pending the collection of new specimens.

Thailand is the most species-rich country of the *Mongolodiaptomus* species, with nine already known taxa plus one new species reported (Ranga Reddy et al. 1998, 2000; Sanoamuang 1999, 2001; Watiroyram and Sanoamuang 2017; Sanoamuang and Watiroyram 2018; Sanoamuang and Dabseepai 2021; this study). Recently, Watiroyram and Sanoamuang (2017) provided a key to the identification of both sexes of the valid *Mongolodiaptomus* species.

During the study of copepod diversity in the forest area of the Plant Genetics Conservation Project in Khon Kaen province, northeast Thailand, we came across a new species of the genus *Mongolodiaptomus*. As a result, this paper deals with the following: i) an illustrated description of *M.phutakaensis* sp. nov.; ii) a review and detailed morphological characteristics comparison of the genus *Mongolodiaptomus*; iii) the interspecies relationships; iv) the biogeography of the genus; and v) the updated key to the genus.

## ﻿Materials and methods

The study area, Kok Phutaka community forest, is located in Wiang Kao District, 78 kilometers from the center of Khon Kaen Province in northeastern Thailand. Her Royal Highness Princess Maha Chakri Sirindhorn of Thailand initiated the Plant Genetics Conservation Project in 1992, which encompasses an area of approximately 1,150 square kilometers. This protected area is a dry dipterocarp forest that provides a source for researchers to study the biodiversity and utilization of plants, animals, and microorganisms. There is one natural swamp and four small artificial ponds in the forest.

Monthly sampling campaigns were conducted from January to December 2007 in all the five above-mentioned habitats using a plankton net with a mesh size of 60 μm. All samples were preserved in 70% ethanol immediately after collection. Specimens were put in a mixture of glycerol and 70% ethanol (ratio 1:10 v/v) and pure glycerol, respectively, just before dissection. Specimens were dissected and mounted at 40–100× magnification under an Olympus SZ51 stereomicroscope. An Olympus compound microscope (CX31) was used to examine all appendages and body ornamentation at 1,000× magnification. All the drawings were created using an Olympus U-DA drawing tube and a compound microscope configured for 100× magnification. Final versions of the drawings were made using the CorelDRAW® 12.0 graphic program. Specimens for scanning electron microscopy (SEM) were dehydrated in an ethanol series (50%, 70%, 80%, 90%, 95%, 100%), for 15 min at each concentration. Specimens were dried in a critical point dryer and coated with gold in a sputter coater. The SEM photographs were taken using a scanning electron microscope (LEO, 1450VP).

The following abbreviations can be found in both the text and the figures: **ae**, aesthetasc; **Enp**, endopod; **Exp**, exopod; **Exp/Enp-n**, exopodal segment n/endopodal segment n; **Pdg1–Pdg5**, pedigers 1–5; **P1–P5**, legs 1–5; **sp**, spine. The nomenclature and descriptive terminology follow Huys and Boxshall (1991), including the analysis of caudal setae (**I–VII**). Type specimens were placed at the Thailand Natural History Museum (**THNHM**) and the Applied Taxonomic Research Center at Khon Kaen University, Thailand (**KKU**).

## ﻿Taxonomic section

**Order Calanoida Sars, 1903**

**Infraorder Neocopepoda Huys & Boxshall, 1991**

**Family Diaptomidae Baird, 1850**

**Sub-family Diaptominae Kiefer, 1932**

**Genus *Mongolodiaptomus* Kiefer, 1937**

### ﻿ Mongolodiaptomus phutakaensis sp. nov.

Taxon classificationAnimaliaCalanoidaDiaptomidae

F96BDEB9-2062-5D05-B7B2-DFAD36A88F81

https://zoobank.org/9B4A5018-E0E5-45C6-9701-A299D8301A81

Figs 1 , 2 , 3 , 4 , 5 , 6 , 7 , 8

Mongolodiaptomus sp. Sanoamuang and Dabseepai (2021): 7, 18, 20.

#### Type locality.

A natural swamp in Kok Phutaka community forest, Muang Kao Phatthana Subdistrict, Wiang Kao District, Khon Kaen Province, northeast Thailand (16°38'43.77"N, 102°18'11.90"E); elevation 220 m a.s.l., water temperature 31.2 °C, pH 8.2, conductivity 299 µS cm^-1^.

#### Type material.

***Holotype***: adult male (THNHM-1V-19371), dissected and mounted in glycerol on one slide. ***Allotype***: adult female (THNHM-1V-19372), dissected and mounted in glycerol on one slide. ***Paratypes***: three adult males and three adult females (THNHM-1V-19373), undissected and preserved in 4% formalin; collected from the type locality on the same date as the holotype. All specimens were collected on 16 August 2007, by P. Dabseepai and K. Koompoot.

#### Description of adult male.

**Figure 1.**
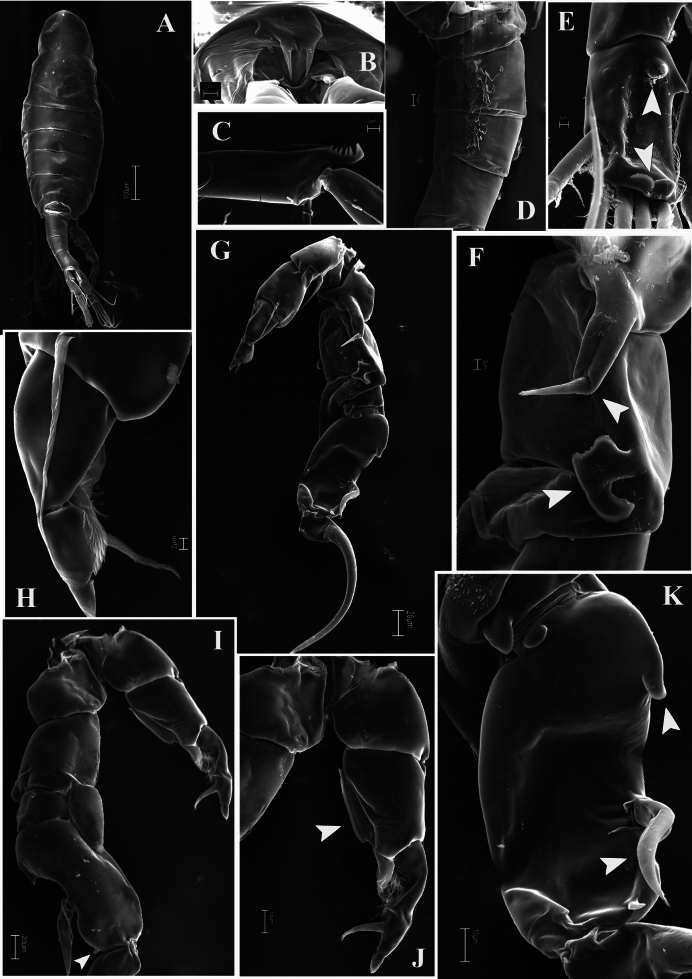
*Mongolodiaptomusphutakaensis* sp. nov., SEM photographs of male **A** habitus, dorsal view **B** rostrum **C** comb-like process on the antepenultimate segment of the right antennule **D** genital somite, and urosomites 2 and 3 **E** right caudal ramus, ventral view (white arrows indicate proximal chitinous spine and distal knob) **F** right P5 coxa and basis, posterior view (white arrows point to the coxal spine and spur-like hyaline membrane) **G** P5 in posterior view **H** distal part of left P5, posterior view **I** P5, anterior view (without end claw, white arrow points to the distal accessory spine) **J** left P5, anterior view (white arrow points to the hyaline membrane) **K** right P5 Exp-1 and 2, posterior view (white arrows point to the proximal accessory spine and twisted principal lateral spine).

**Figure 2.**
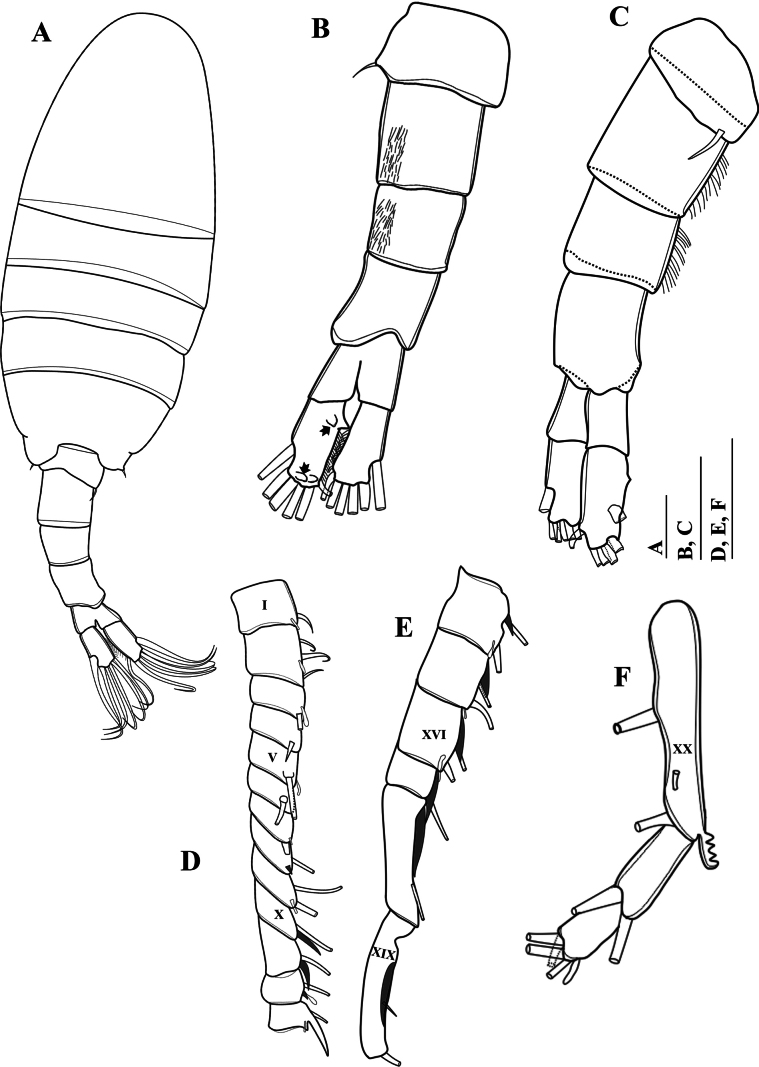
*Mongolodiaptomusphutakaensis* sp. nov., male **A** habitus, dorsal view **B** urosome and caudal rami, ventral view (black arrows indicate chitinous spine and knob on right caudal ramus) **C** urosome and caudal rami, lateral view **D–F** right antennule **D** segments 1–13 **E** segments 14–19 **F** segments 20–22. Scale bar: 100 µm.

Total body length, measured from anterior margin of rostrum to posterior margin of caudal rami, 1.3–1.4 mm (mean = 1.37 mm, *n* = 10), (Figs 1A, 2A). Body smaller and slender than in female. Prosome ~ 2.2 × as long as urosome (Fig. 2A). Rostrum (Fig. 1B) well developed, with two spiniform processes. Pedigers 4 and 5 fused except at lateral margins. Lateral wings of Pdg 5 asymmetrical; right postero-lateral wing shorter than left one; each wing with one thin postero-lateral spine (Fig. 2A).

Urosome (Figs 1D, 2A–C) with five somites. Genital somite dilated postero-laterally on right side, shorter than wide, with a curved spine on the right posterolateral corner. Urosomites 2–4 approximately as long as wide each. Urosomites 2–3 (Figs 1D, 2B, C) with a patch of hairs on right ventral side. Urosomite 4 with expanded right dorso-posterior margin. Anal somite asymmetrical, right side slightly longer than left side (Fig. 2A, C). Caudal rami asymmetrical (Figs 1E, 2A–C), each ramus ~ 2.3 × as long as wide, inner right margin hairy (Figs 1E, 2B). Right ramus armed with four chitinous structures on ventral surface; two sharp tips situated proximally and two semicircular knobs distally (Figs 1E, 2B). Each ramus with six setae (setae II–VII): setae II–VI plumose, anterolateral (II) seta with smooth region on outer margin proximally; terminal setae (setae IV and V) without fracture plane; dorsal seta (VII) articulated, bare, longest.

**Figure 3.**
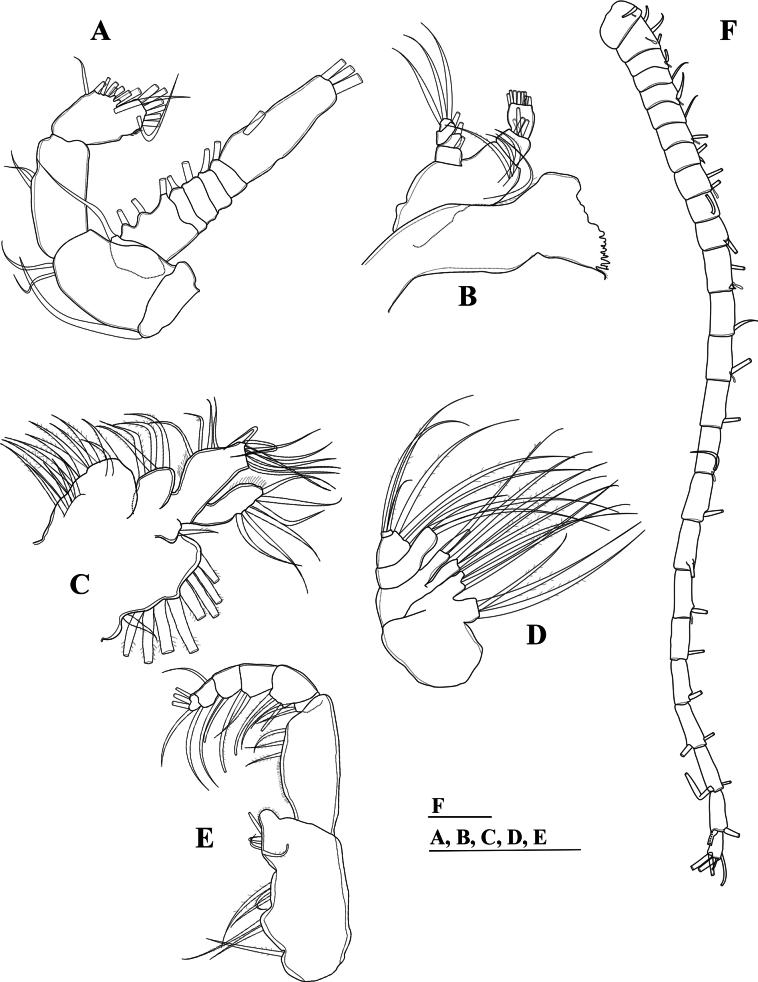
*Mongolodiaptomusphutakaensis* sp. nov., male **A** antenna **B** mandible **C** maxillule **D** maxilla **E** maxilliped **F** left antennule. Scale bar: 100 µm.

Antennule: asymmetrical, extending beyond the end of caudal setae. Left antennule (Fig. 3F): 25-segmented. Armature formula as in Table 1. Right antennule (Figs 1C, 2D–F) 22-segmented. Armature formula as in Table 2. External extension on antepenultimate segment (segment XX) short, comb-like, with five or six teeth (Figs 1C, 2F).

**Table 1.** Armature formula of the left male antennule of *Mongolodiaptomusphutakaensis* sp. nov. The number of setae (Arabic numerals), aesthetascs (ae), and spines (sp) is given. The Roman numerals refer to segment numbers.

	Segment number												
	I	II	III	IV	V	VI	VII	VIII	IX	X	XI	XII	XIII
Number of elements	1+ae	3+ae	1+ae	1	1+ae	1	1+ae	1+sp	2+ae	1	1	1+ae+sp	1
	**XIV**	**XV**	**XVI**	**XVII**	**XVIII**	**XIX**	**XX**	**XXI**	**XXII**	**XXIII**	**XXIV**	**XXV**	
Number of elements	1+ae	1	1+ae	1	1	1+ae	1	1	2	2	2	4+ae	

**Table 2.** Armature formula of the right male antennule of *Mongolodiaptomusphutakaensis* sp. nov. The number of setae (Arabic numerals), aesthetascs (ae), and spines (sp) is given. The Roman numerals refer to segment numbers.

	Segment number										
	I	II	III	IV	V	VI	VII	VIII	IX	X	XI
Number of elements	1+ae	3+ae	1+ae	1	1+ae	1	1+ae	1+sp	2+ae	1+sp	1+sp
	**XII**	**XIII**	**XIV**	**XV**	**XVI**	**XVII**	**XVIII**	**XIX**	**XX**	**XXI**	**XXII**
Number of elements	1+ae+sp	1+ae+sp	2+ae+sp	2+ae+sp	2+ae+sp	1+sp	1+sp	2+sp	3+sp	2	4+ae

Antenna (Fig. 3A): coxa and basis with one and two bare setae on inner distal corner, respectively. Enp two-segmented; Enp-1 with two setae along inner margin; Enp-2 with nine setae along inner margin, seven setae apically; all setae bare. Exp seven-segmented: Exp-1–6 with 1, 3, 1, 1, 1, 1 setae along inner margin; Exp-7 with one seta on inner margin and three setae apically; all setae bare.

Mandible (Fig. 3B): ~ 6 cuspidate teeth dorsally and one seta on coxal gnathobase dorsally. Basis with four bare setae: one proximally and three distally along inner margin. Enp-1 with four setae on inner distal corner. Enp-2 with nine setae apically; two oblique rows of spinules along outer margin. Exp-1–3 each with one seta on inner margin; Exp-4 with three setae apically; all setae bare.

Maxillule (Fig. 3C): praecoxal arthrite with nine strong setae laterally and four slender submarginal setae. Coxal endite with four setae; coxal epipodite with nine setae; two proximal-most setae smaller than others. Two basal endites fused to segment bearing them: proximal and distal endite, each with four setae apically; basal exopodite with one short seta. Enp-1 and Enp-2 each with four setae apically, proximal segment fused to basis. Exp with six bare setae apically.

Maxilla (Fig. 3D): praecoxa fused to coxa. Proximal and distal endites on praecoxa with three setae apically each. Two coxal endites with three setae apically each. Allobasis with three setae apically. Enp two-segmented; with three setae each.

Maxilliped (Fig. 3E): four medial lobes on syncoxa: setal formula 1, 2, 3, 4, respectively; subdistal inner margin produced into a spherical lobe with a patch of tiny spinules. Basis with three setae along medial inner margin, with a row of tiny spinules proximately. Enp six-segmented, with 2, 3, 2, 2, 2, and 4 bare setae, respectively.

**Figure 4.**
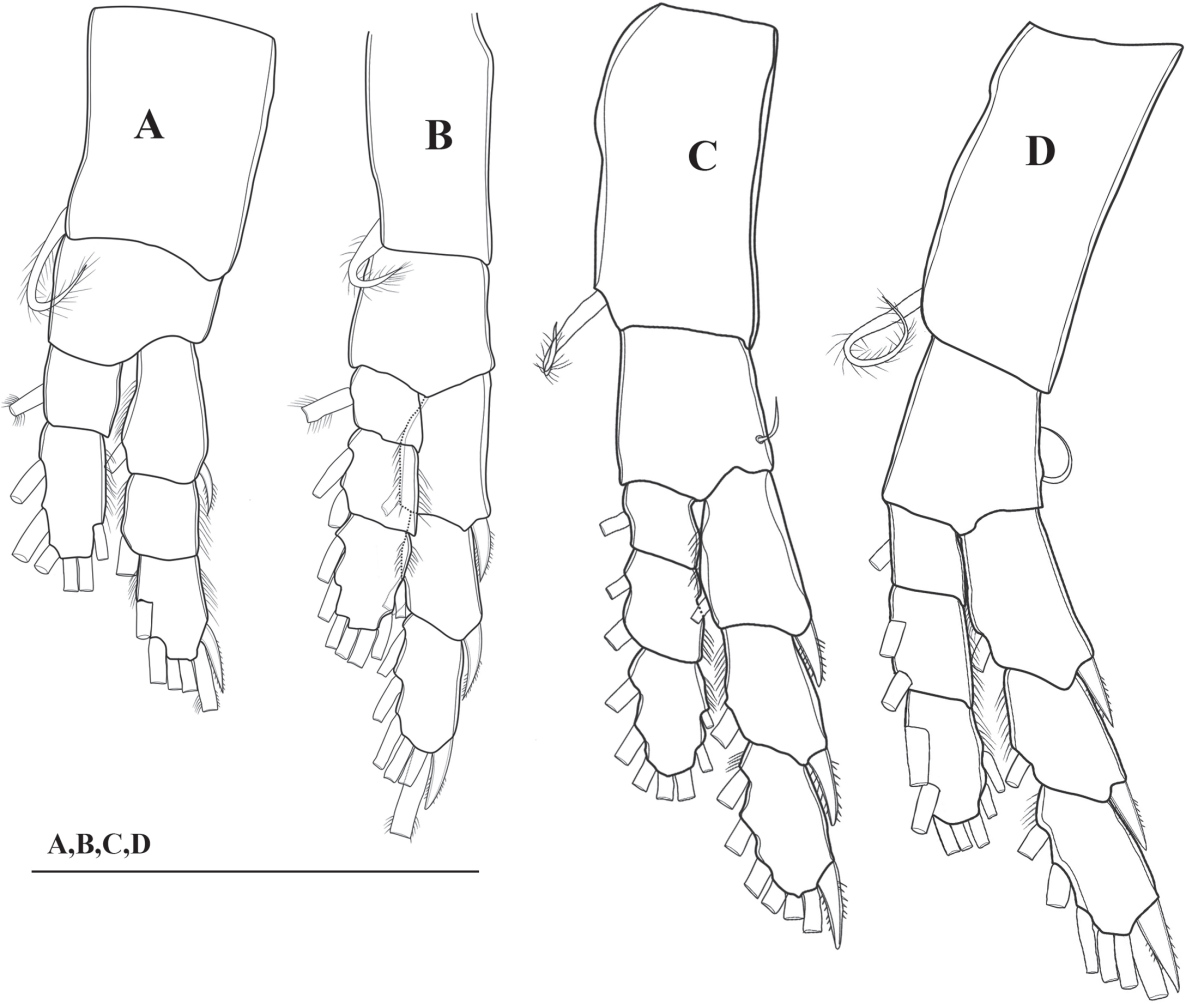
*Mongolodiaptomusphutakaensis* sp. nov., male **A** P1 **B** P2 **C** P3 **D** P4. Scale bar: 100 µm.

P1–P4 (Fig. 4A–D): coxa with a pinnate seta at innermost distal corner. P1 and P2 basis without setae; a reduced bare seta on outer distal margin of P3 and P4. Exp longer than Enp; two-segmented Enp and three-segmented Exp on P1, three-segmented Enp and Exp on P2–P4. Armature formula of P1–P4 as in Table 3.

**Table 3.** Armature formula of the swimming legs of *Mongolodiaptomusphutakaensis* sp. nov. The number of setae (Arabic numerals) and spines (Roman numerals) is given in the following sequence: outer-inner margin or outer-apical-inner margin.

	Coxa	Basis	Exp			Enp		
			1	2	3	1	2	3
P1	0–1	0–0	I–1	0–1	I–3–2	0–1	1–2–3	----
P2	0–1	0–0	I–1	I–1	I–3–3	0–1	0–2	2–2–3
P3	0–1	1–0	I–1	I–1	I–3–3	0–1	0–2	2–2–3
P4	0–1	1–0	I–1	I–1	I–3–3	0–1	0–2	2–2–3

**Figure 5.**
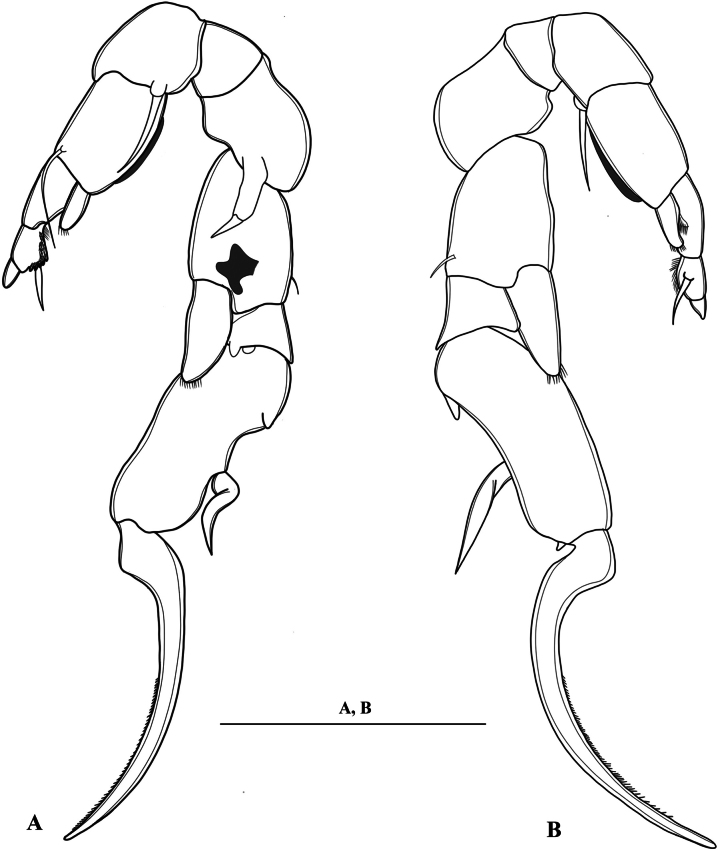
*Mongolodiaptomusphutakaensis* sp. nov., male **A** P5, posterior view **B** P5, anterior view. Scale bar: 200 µm.

P5 (Figs 1F–K, 5A, B): intercoxal sclerite trapezoidal, inner distal margin not produced, without any projection. Right P5: coxa with an acute, robust spine on extension on posterior surface, its tip bent inward (Figs 1F, 5A), inner distal margin slightly produced into a rounded lobe. Basis rectangular, ~ 1.5 × as long as wide; with prominent irregular-shaped chitinous process at mid-distal length on posterior surface (Figs 1F, 5A); a small seta on distal outer margin; without any hyaline structure. Enp one-segmented, gradually tapering to distal end, tipped with tiny spinules distally; reaching beyond 1/3 of Exp-2. Exp-1 shorter than wide, with two chitinous knobs at distal inner corner; outer distal margin produced into acute tip (Figs 1I, 5A, B). Exp-2 slightly incurved, inner margin convex, outer margin concave, ~ 2.5 × as long as wide, with two small processes proximally and distally; principal lateral spine inserted slightly posterior to mid-length of outer margin. Principal lateral spine (Figs 1K, 5A, B) somewhat slightly curved, robust, ~ 1/2 length of segment. Accessory lateral spine (Figs 1K, 5A, B) minute, distal spine situated close to insertion of end-claw (Figs 1I, 5B), distal accessory spine smaller than proximal one. End-claw sickle-shaped, long, and slender, with a serrate inner margin, with blunt tip; ~ 1.5 × as long as Exp-2.

Left P5 (Figs 1G–J, 5A, B): coxa with long bare seta on posterior lobe near distal inner corner; longer and slender than spine on right coxal segment. Basis with long narrow hyaline lamella along inner margin (Figs 1J, 5A, B); slender, long posterolateral seta on posterior surface, reaching to middle of Exp-2 segment (Figs 1H, 5A, B). Exp-1 trapezoidal, tapering towards distal end, medial margin concave with a field of setules (Figs 1H, 5A, B). Exp-2 smaller than Exp-1, with inner robust seta, longer than Exp-2; with inner strongly serrate margin (Figs 1H, 5A, B). Exp-3 reduced to thumb-like segment. Enp one-segmented, shorter than Exp-1, with spinulated tip.

#### Description of adult female.

**Figure 6.**
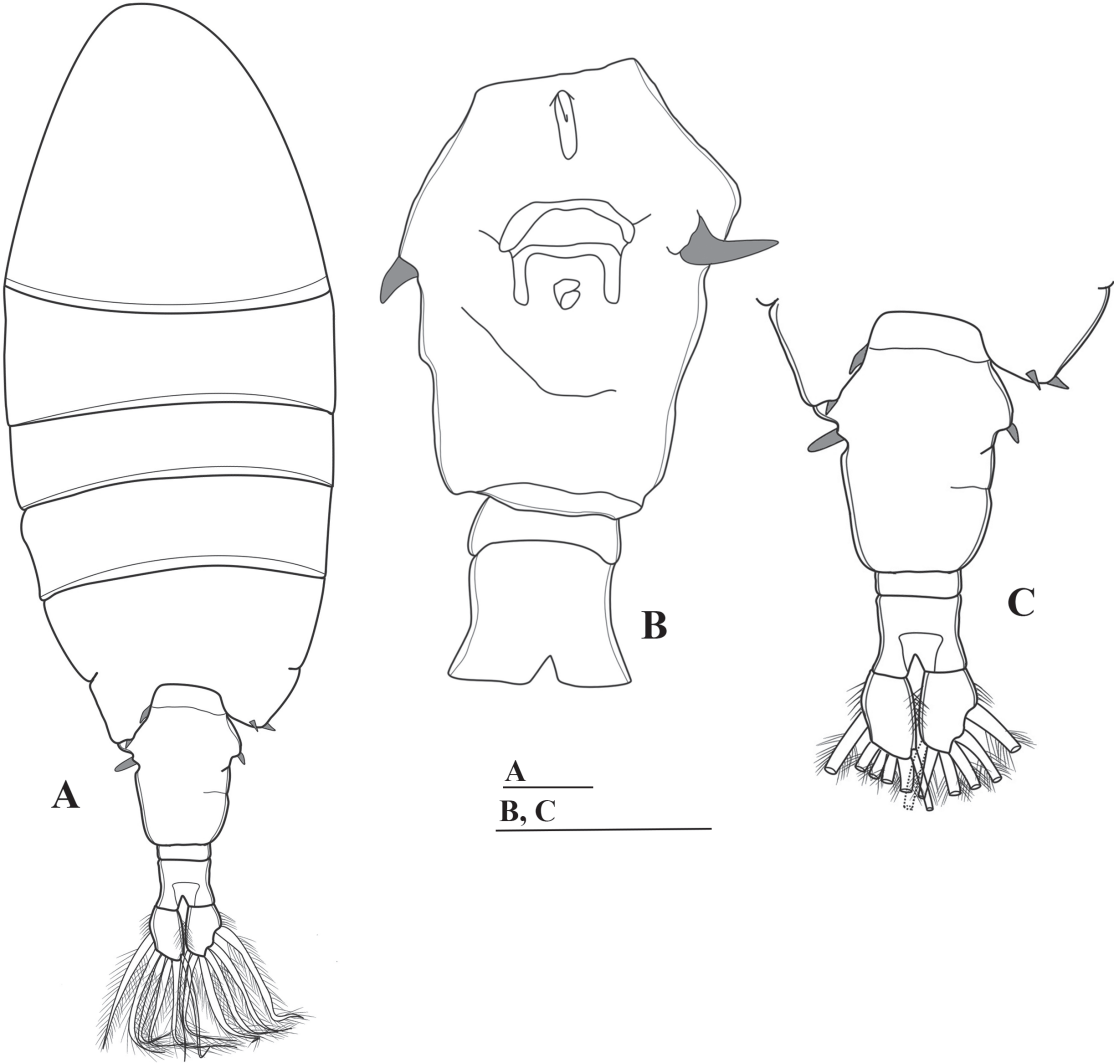
*Mongolodiaptomusphutakaensis* sp. nov., female **A** habitus, dorsal view **B** urosome, ventral view (without caudal rami) **C** pediger 5, urosome, and caudal rami, dorsal view. Scale bar: 100 µm.

Total body length, measured from anterior margin of rostrum to posterior margin of caudal rami, 1.5–1.7 mm (mean = 1.6 mm, *n* = 10) (Figs 6A, 8A). Prosome: urosome ratio ~ 2.4:1. Prosome similar to that of male. Rostrum fused, symmetrical, acutely pointed (Fig. 8B). Fourth and fifth pedigerous somites incompletely fused. Fifth pediger with sub-asymmetrical posterolateral wings (Figs 6A, C, 8C); right wing rounded, left wing triangular and longer than right wing. Urosome 3-segmented, with asymmetrical genital double-somite (Fig. 6A–C). Genital double-somite longer than urosomite 2, anal somite, and caudal rami combined (Figs 6B, C, 8C); right proximal region slightly curved with small spine. Left side with large dorsolateral spine on sub-proximal region. A pair of gonopores and copulatory pores located centrally at ~ 1/2 length of genital double-somite (Figs 6B, 8H). Urosomite 2 symmetrical, shorter than wide. Anal somite symmetrical, as long as length of caudal rami (Figs 6C, 8I); anal operculum small with convex free margin. Caudal rami parallel, symmetrical; both rami with hairy inner and outer margins (Figs 6C, 8I). All principal caudal setae slightly dilated anteriorly; dorsal seta approximately as long as principal setae.

Antennule symmetrical; left antennule, antenna, mouthparts, and P1–P4 as in male.

**Figure 7.**
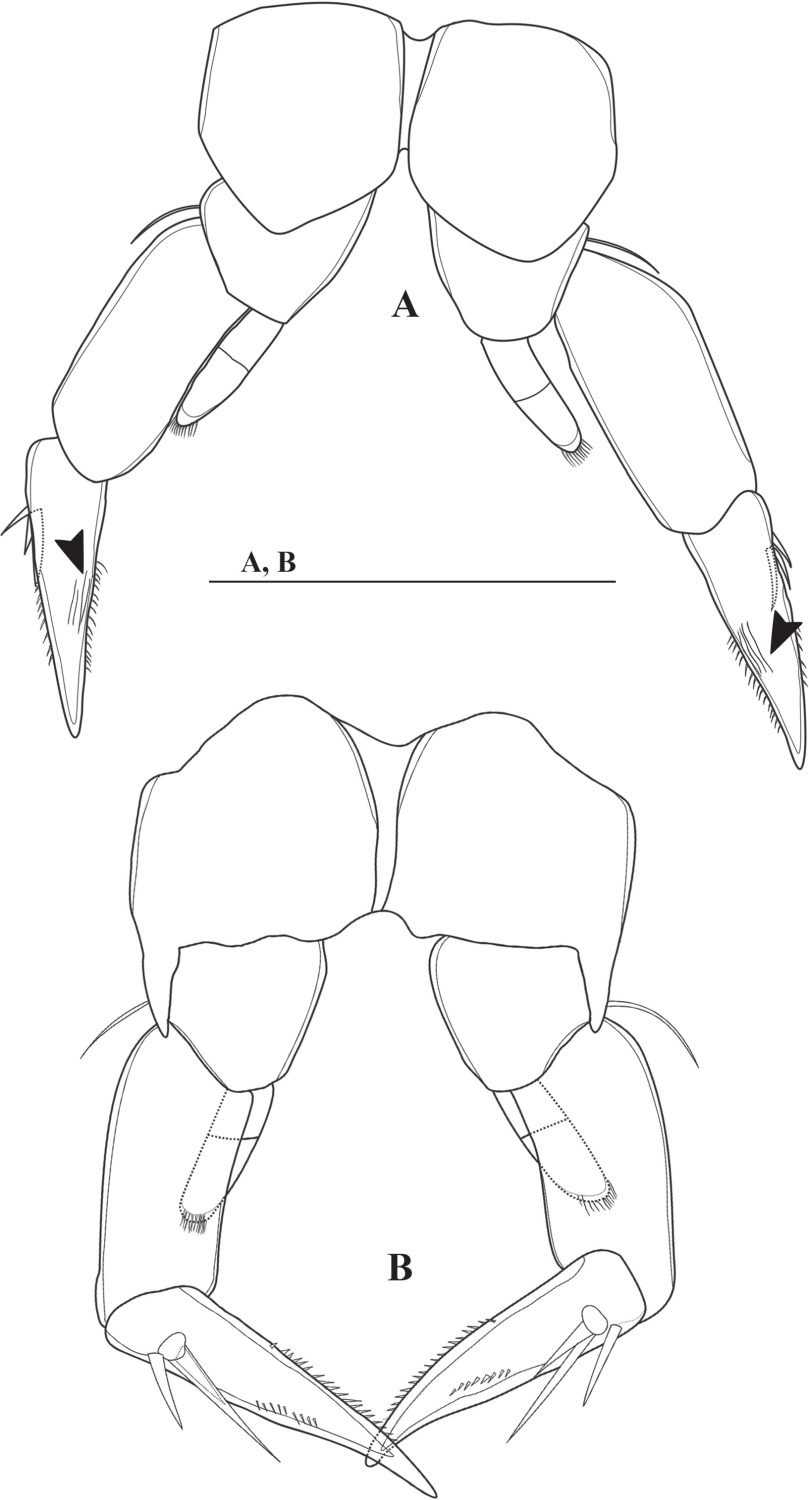
*Mongolodiaptomusphutakaensis* sp. nov., female. P5 **A** P5, posterior view (black arrows indicate longitudinal ridges) **B** P5, anterior view. Scale bar: 100 µm.

**Figure 8.**
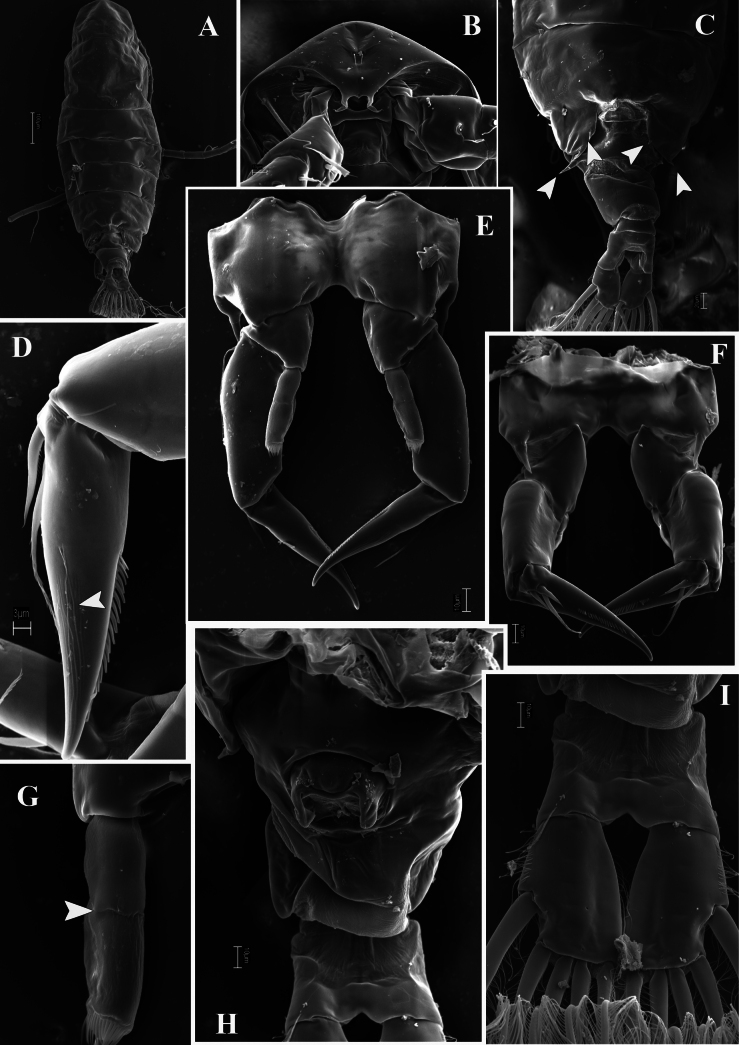
*Mongolodiaptomusphutakaensis* sp. nov., SEM photographs of female **A** habitus, dorsal view **B** rostrum **C** pediger 5 and urosome, dorsal view (white arrows point to spines) **D** P5 Exp-1–2, posterior view (white arrow indicates longitudinal ridges) **E** P5, posterior view **F** P5, anterior view **G** P5 Enp, anterior view (white arrow indicates the border of the two segments) **H** urosome, ventral view **I** caudal rami, ventral view.

P5 symmetrical (Figs 7A, B, 8D–G). Intercoxal sclerite narrow, triangular. Distal outer margin of coxa extended on anterior side into spiniform apophysis reaching distal part of Exp-1 (Fig. 7B). Basis with thin, bare seta on outer margin, reaching ~ 1/4 of Exp-1 length. Exp three-segmented (Figs 7A, B, 8E, F). Exp-1 sub-rectangular, ~ 2.3 × as long as wide. Exp-2 triangular, with a row of strong spinules along both margins; with longitudinal grooves (conveyor canals) on posterior view (Figs 7A, 8D), small outer spine proximally. Exp-3 reduced, represented by a small segment on proximal outer margin of Exp-2, armed with two unequal spiniform setae apically. Enp two-segmented (Fig. 8G), subconical, ~ 2/3 as long as Exp-1; with obliquely truncate and finely spinulose apex.

#### Etymology.

The specific epithet is derived from Kok Phutaka, reflecting the name of the area in which the type locality is located. The name is an adjective in the nominative singular, gender feminine.

#### Distribution.

At present, the new species has been found only in the type locality, a natural swamp in Kok Phutaka community forest in Khon Kaen Province, northeast Thailand. It co-exists with other calanoids, *Phyllodiaptomuspraedictus* Ranga Reddy & Dumont, 1994 and *Mongolodiaptomusrarus* (Ranga Reddy, Dumont, & Sanoamuang, 1998). The other artificial ponds nearby also contained *M.botulifer* (Kiefer, 1974). Representatives of the new species were observed only once out of approximately 5,000 sampled sites throughout Thailand. Currently, this species is endemic to Thailand. The new species is present in only one locality throughout the year, and ecological parameters varied in a temperature range of 25.0–32.5 °C (mean = 29.13 °C), conductivity 74–495 µS cm^-1^ (mean = 201.25 µS cm^-1^), and pH 5.16–10.03 (mean = 7.66).

### ﻿Key to the species of *Mongolodiaptomus* Kiefer, 1937

**Males**:

**Table:** 

1	Spinous process on antepenultimate segment of right antennule is slender and smooth	**2**
–	Spinous process on antepenultimate segment of right antennule is serrated or comb-like	**4**
2	Right P5 Enp conical, proximal part very broad and swollen, distal part tapering, extended to 1/3 length of inner margin of Exp-2	***M.birulai***
–	Right P5 Enp large, conical, reaching to nearly 3/4 length of inner margin of Exp-2	**3**
3	Right P5 basis with triangular hyaline membrane on inner margin	***M.malaindosinensis***
–	Right P5 basis with spherical hyaline membrane on inner margin	***M.botulifer***
4	P5 intercoxal plate with outgrowth on distal margin	**5**
–	P5 intercoxal plate without outgrowth on distal margin	**8**
5	Principal lateral spine on right P5 Exp-2 straight	**6**
–	Principal lateral spine on right P5 Exp-2 curved and twisted	**7**
6	P5 intercoxal plate with rounded lobe on distal margin	***M.pectinidactylus***
–	P5 intercoxal plate with spine-like lobe on distal margin	***M.uenoi***
7	Spine-like process on P5 intercoxal plate with 1 strong spine	***M.mekongensis***
–	Spine-like process on P5 intercoxal plate with 2 strong spines	***M.loeiensis***
8	Right P5 basis with hyaline membrane on inner margin	***M.mephistopheles***
–	Right P5 basis without hyaline membrane on inner margin	**9**
9	Left P5 basis with hyaline membrane on inner margin	**10**
–	Left P5 basis without hyaline membrane on inner margin	**11**
10	Principal lateral spine on right P5 Exp-2 straight	***M.dumonti***
–	Principal lateral spine on right P5 Exp-2 bent	***M.calcarus***
–	Principal lateral spine on right P5 Exp-2 bent and twisted	***M.phutakaensis* sp. nov.**
11	Right P5 basis with chitinous spur on posterior surface	***M.rarus***
–	Right P5 basis without any process on posterior surface	***M.gladiolus***

**Females**:

**Table:** 

1	P5 Enp 1-segmented	**2**
–	P5 Enp 2-segmented	**7**
2	Genital double-somite with postero-laterally oriented conical outgrowth on proximal right side	**3**
–	Genital double-somite without postero-laterally oriented outgrowth on proximal right side	**6**
3	Left spine inserted on lobe-like process of genital double-somite	***M.gladiolus***
–	Left spine inserted directly on genital double-somite	**4**
4	Genital double-somite with expanded right distal corner	**5**
–	Genital double-somite without expanded right distal corner	***M.uenoi***
5	P5 with long Enp, reaching beyond distal end of Exp-1	***M.malaindosinensis***
–	P5 with short Enp, not reaching distal end of Exp-1 (2/3 of Exp-1 length)	***M.botulifer***
6	Genital double-somite with longer spine on left side compared to right side	***M.mephistopheles***
7	P5 Exp-3 absent	***M.birulai***
–	P5 Exp-3 present	**8**
8	Genital double-somite with postero-laterally oriented outgrowth on proximal right side	**9**
–	Genital double-somite without postero-laterally oriented outgrowth on proximal right side	**11**
9	Genital double-somite with hyaline membrane along inner margin on right side	***M.rarus***
–	Genital double-somite without hyaline membrane along inner margin on right side	**10**
10	Lateral wings on Pdg 5 (left: right) symmetrical	***M.loeiensis***
–	Lateral wings on Pdg 5 (left: right) asymmetrical	***M.mekongensis***
11	Spine on left side of genital double-somite similar in size to spine on right side	***M.pectinidactylus***
–	Spine on left side of genital double-somite larger than spine on right side	**12**
12	Genital double-somite somewhat rectangular in shape	***M.dumonti***
–	Genital double-somite with swollen proximal part and distal part tapering to end	**13**
13	Lateral wings on Pdg 5 (left: right) sub-symmetrical	***M.calcarus***
–	Lateral wings on Pdg 5 (left: right) asymmetrical	***M.phutakaensis* sp. nov.**

## ﻿Discussion

*Mongolodiaptomusphutakaensis* sp. nov. exhibits the distinguishing features of the genus, as described in the updated generic traits outlined by Ranga Reddy et al. (2000). For the males, the right P5 Exp-2 carries the characteristic three lateral spines, one principal spine inserted at the middle of the segment on the outer margin, and two accessory spines located proximally and distally. A comparison with its congeners shows that *M.phutakaensis* sp. nov. resembles the two recently described taxa from the Mekong region: *M.loeiensis* Watiroyram & Sanoamuang, 2017 and *M.mekongensis* Sanoamuang & Watiroyram, 2018, respectively. These three closely related species can be distinguished from the other congeners by the unique shape of the male right P5 Exp-2; the inner margin is slightly incurved, the proximal and distal parts of the outer margin are enlarged, and the principal lateral spine is bent and twisted. The antepenultimate segment of the male right antennule of all three related species has a comb-like process. Other similarities among the three closely related species are the male right P5 has a spur-like or irregular process on the basis, the coxa has a strong spine, the Exp-1 has an acute process on the outer distal margin, the left P5 has a hyaline lamella on the inner margin of the basis, and the strong spinules along the inner margin of the Exp-2. The female of the new species shares similarities with *M.loeiensis* and *M.mekongensis* by having two-segmented P1 Enp.

The new species can be differentiated from *M.mekongensis* and *M.loeiensis* by the characteristics of the male caudal rami and P5 (Table 4): the right caudal ramus of *M.phutakaensis* sp. nov. has four ventral chitinous processes (two proximal spine-like processes and two distal semi-circular knobs), while *M.mekongensis* has one spine-like process and one semi-circular knob, and *M.loeiensis* has two spine-like processes and one semi-circular knob. The intercoxal plate of the new species is slightly produced distally and without any spine, but it is well-produced with one strong spine and two spines on its distal margin in *M.mekongensis* and *M.loeiensis*, respectively. The right P5 basis in males lacks a hyaline membrane on the inner margin in the new species, which is present in *M.mekongensis* and *M.loeiensis*. The male P5 has a distinct mid-distal spur-like chitinous process on the posterior surface in the new species versus a small chitinous prominence on the same position in *M.mekongensis* and *M.loeiensis*. The left P5 basis has a thin, longer hyaline lamella on the inner margin in the new species but is somewhat shorter in *M.mekongensis* and *M.loeiensis*. Only *M.mekongensis* has an obviously longitudinal chitinous ridge on the posterior side of this segment.

**Table 4.** The morphological characteristics and distribution of the closely related *Mongolodiaptomus* species: *M.loeiensis*, *M.mekongensis*, and *M.phutakaensis* sp. nov.

Characters and distribution	*M.loeiensis*	*M.mekongensis*	*M.phutakaensis* sp. nov.
**MALE**			
Chitinous teeth on ventral surface of the right caudal ramus	Two	One	Two
Chitinous (semicircular) knob on ventral surface of the right caudal ramus	One	One	Two
Spine-like process on the P5 intercoxal plate	Two strong spines	One strong spine	Absent
Right P5 basis with inner hyaline membrane	Yes	Yes	No
Left P5 basis with longitudinal chitinous ridge	No	Yes	No
Left P5 basis with extra-long posterolateral seta on posterior surface (longer than Exp-1 segment)	No	No	Yes
**FEMALE**			
Lateral wings on Pdg 5 (left: right)	Symmetrical	Asymmetrical	Asymmetrical
Right side of genital double-somite with well-developed posterolateral process	Yes	Yes	No
P5 Exp-2 with longitudinal grooves (conveyor canals) on posterior view	No	Yes	Yes
**DISTRIBUTION**	Thailand endemic (a temporary pond in Loei Province, northeast Thailand)	Mekong region (temporary-water habitats in northeast Thailand, Laos, Cambodia, Vietnam, and South China)	Thailand endemic (a natural swamp in Khon Kaen Province, northeast Thailand)

In the females, the left wing of Pdg 5 is longer than the right one in the new species and *M.mekongensis*, whereas both wings are symmetrical in *M.loeiensis*. The genital double-somite is only slightly asymmetrical in the new species but pronounced in *M.mekongensis* and *M.loeiensis*, with well-developed posterolateral outgrowth on the right side. The P5 Exp-2 has longitudinal grooves (conveyor canals) on the posterior view in the new species and *M.mekongensis*, but these grooves are absent in *M.loeiensis*.

### ﻿Review of taxonomic characters of *Mongolodiaptomus* species

Currently, 13 species of the genus *Mongolodiaptomus* have been reported worldwide (see Table 2 in Sanoamuang and Watiroyram 2018; Sanoamuang and Dabseepai 2021; this study). This number does not include *Mongolodiaptomusmariadvigae* (Brehm, 1921) and *M.formosanus* Kiefer, 1937. According to Li et al. (2018) and Walter and Boxshall (2024), *M.mariadvigae* has been transferred to *Neutrodiaptomusmariadvigae* (Brehm, 1921). For the status of *M.formosanus*, several scientists from China (Shen et al. 1979; Li et al. 2018), Taiwan (Young and Shih 2011; Young et al. 2013), and Vietnam (Tran et al. 2016) considered it a synonym of *M.birulai*. Thus, in this paper, we treat *M.mariadvigae* as a member of *Neutrodiaptomus* and *M.formosanus* as a synonym of *M.birulai*. Another doubtful taxon, *M.malaindosinensis*, is considered a synonym of *M.botulifer* by Ranga Reddy et al. (2000), but after detailed examinations of specimens from Thailand, Cambodia, and Vietnam, we considered *M.malaindosinensis* as a distinct species (Sanoamuang 2002; Watiroyram and Sanoamuang 2017; Boonmak et al. 2018; Sanoamuang and Watiroyram 2018, 2023; Sanoamuang and Dabseepai 2021; Boonmak and Sanoamuang 2022; Chaicharoen and Sanoamuang 2022).

A comparison of male and female morphological characters of the *Mongolodiaptomus* species is presented in Tables 5–6. The prominent morphological characteristics of this genus are reviewed briefly hereafter.

**Table 5.** Comparison of male morphological characters of *Mongolodiaptomus* species (? means unknown or doubtful).

Male characters	*M.birulai*	*M.botulifer*	*M.malaindosinensis*	*M.gladiolus*	*M.calcarus*	*M.rarus*	*M.dumonti*	*M.mephistopheles*	*M.uenoi*	*M.pectinidactylus*	*M.loeiensis*	*M.mekongensis*	*M.phutakaensis*
**Right antennule**													
- segment 16 with spine		+		+	+		+	+	+			+	+
- segment 16 without spine	+		+			+				+	+		
- spinous process on antepenultimate segment long and slender	+	+	+										
- spinous process on antepenultimate segment comb-like				+	+	+	+	+	+	+	+	+	+
**Urosomites 2 and 3**													
- with ventral hairs	+	+	+	?	+		+		+	+	+	+	+
- without ventral hairs				?		+		+					
**Right caudal ramus**													
- with chitinous structure ventrally	+	+	+		+	+	+	+	+		+	+	+
- without chitinous structures ventrally				+						+			
**Right P5**													
- intercoxal plate produced	+	+	+						+	+	+	+	
- intercoxal plate unproduced				+	+	+	+	+					+
- basis with inner hyaline lamella	+	+	+			+		+	+	+	+	+	
- basis without inner hyaline lamella				+	+		+						+
- basis with spurlike process					+	+	+			+	+	+	+
- basis without spurlike process	+	+	+	+				+	+				
- Exp-1 with pointed spinous process at distal outer corner		+	+					+	+		+	+	+
- Exp-1 with blunt spinous process at distal outer corner	+			+	+	+	+			+			
- principal lateral spine of Exp-2 located at or close to mid-length of outer margin	+	+	+	+	+	+		+	+	+	+	+	+
- principal lateral spine of Exp-2 located at ¾ length of outer margin							+						
- principal lateral spine of Exp-2 straight				+		+	+		+	+			
- principal lateral spine of Exp-2 curved	+	+	+		+			+					
- principal lateral spine of Exp-2 curved and twisted											+	+	+
- Enp: obovate shaped, ~ ¾ length of Exp-2 segment		+	+										
- Enp: conical shaped, ≤ ½ length of Exp-2 segment	+			+	+	+	+	+	+	+	+	+	+
**Left P5**													
- basis with inner hyaline lamella or knoblike outgrowth	+	+	+		+	+	+	+	+	+	+	+	+
- basis without inner hyaline lamella				+									
- Enp one-segmented	+			+	+	+	+	+	+	+	+		+
- Enp two-segmented		+	+									+	

**Table 6.** Comparison of female morphological characters of *Mongolodiaptomus* species (? means unknown or doubtful).

Female characters	*M.birulai*	*M.botulifer*	*M.malaindosinensis*	*M.gladiolus*	*M.calcarus*	*M.rarus*	*M.dumonti*	*M.mephistopheles*	*M.uenoi*	*M.pectinidactylus*	*M.loeiensis*	*M.mekongensis*	*M.phutakaensis*
**Lateral wings on Pdg 5 (left: right)**													
- symmetrical					+		+			+	+		
- asymmetrical	+	+	+	+		+		+	+			+	+
**Genital double-somite**													
- right proximal region with well-developed posterolateral process	+	+							+		+	+	
- right proximal region with moderately developed posterolateral process			+	+		+				+			+
- right proximal region without posterolateral process					+		+	+					
- right distal corner expanded		+	+				+						
- right distal corner not expanded	+			+	+	+		+	+	+	+	+	+
**P5**													
- seta on basis longer than ½ length of Exp-1		+	+		+	+	+	+	+	+	+	+	
- seta on basis shorter than ½ length of Exp-1	+			+									+
- Exp-3 inarticulate (fused with Exp-2)		+		+					+				
- Exp-3 distinct	?		+		+	+	+	?		+	+	+	+
- Enp one-segmented		+	+	+				+	+				
- Enp two-segmented	+				+	+	+			+	+	+	+

#### ﻿Antennule

While the setal armature of the female antennules remains conservative among species, the characteristics of the male grasping antennules serve to identify species. The degree of spine development on segments 8 and 15 is important at the species level; segment 16 bears a spinous projection in eight species but is absent in five species (Table 5). The spinous process on the antepenultimate segment of most species is comb-like, but it is long and slender in three species (*M.birulai*, *M.botulifer*, and *M.malaindosinensis*). However, the shape and size of the comb-like projections are different across the species (Table 5).

#### ﻿Lateral wings of fifth pediger

In the female, the shape and size of the lateral wings and the position of the inner (posterior) spine on either wing are of significant taxonomic value. In most species, both left and right wings are moderate in size and moderately asymmetrical, only four (*M.calcarus*, *M.dumonti*, *M.loeiensis*, and *M.pectinidactylus*) have symmetrical wings (Table 6).

#### ﻿Urosome

The relative lengths of urosomites and caudal rami, as well as the structural details of the female’s genital double-somite, are highly diagnostic. The genital double-somite’s relative length varies greatly between species. It is strikingly asymmetrical. In five species (*M.birulai*, *M.botulifer*, *M.loeiensis*, *M.mekongensis*, and *M.uenoi*), the right proximal region has a well-developed posterolateral process, while in the other five species (*M.gladiolus*, *M.malaindosinensis*, *M.pectinidactylus*, *M.phutakaensis* sp. nov., and *M.rarus*), it has a moderately developed posterolateral process. In the male, most species have ventral hairs on urosomites 2 and 3, but only *M.mephistopheles* and *M.rarus* do not have ventral hairs on those segments. In general, the male right caudal ramus of most species is armed with one or two chitinous structures and sometimes with two minute semicircular knobs ventrally; only *M.gladiolus* and *M.pectinidactylus* do not have such structures.

#### ﻿Male fifth leg (P5)

The interspecific differences in the male P5 are well pronounced in this genus. On the right and left P5, the shape and structure of the Exp-2 and of the Enp are different in most species (Table 5). On the right P5, the inner coxal plate is uniquely produced in six species, particularly with one and two strong spines in *M.mekongensis* and *M.loeiensis*, respectively. The basis in most species has inner hyaline lamella, except in *M.calcarus*, *M.dumonti*, *M.gladiolus*, and *M.phutakaensis* sp. nov. Furthermore, another distinctive characteristic of the genus is the presence of a spur-like process at the mid-distal margin of the posterior surface of the basis in seven species (Table 5). In seven species, the Exp-1 of the right P5 has a pointed spinous process in the distal outer corner. The principal lateral spine of Exp-2 is located at or close to mid-length of the outer margin in all species except *M.dumonti*, where such a spine is located at 3/4 length of the outer margin. The principal lateral spine of Exp-2 is either straight, curved, or twisted. On the left P5, the basis has either inner hyaline lamella or knoblike outgrowth in all but *M.gladiolus*. The Enp is one-segmented except for *M.botulifer*, *M.malaindosinensis*, and *M.mekongensis*.

#### ﻿Female fifth leg

The seta on basis is longer than ½ length of Exp-1 in most species except *M.birulai*, *M.gladiolus*, and *M.phutakaensis* sp. nov. The Exp-3 is distinct in all but *M.botulifer*, *M.gladiolus*, and *M.uenoi*. The Enp are two-segmented except for *M.botulifer*, *M.gladiolus*, *M.malaindosinensis*, *M.mephistopheles*, and *M.uenoi*.

### ﻿Interspecies relationships

Recently, Sanoamuang and Watiroyram (2018) divided the known species of *Mongolodiaptomus* based on the male characters into three species groups. Hereafter, an amended proposal is presented to include all known species of *Mongolodiaptomus* in four groups:

1. The *birulai* species group includes *M.botulifer*, *M.birulai*, and *M.malaindosinensis* and exhibits the following characteristics: (1) the spinous process on the antepenultimate segment of the right antennule is slender and smooth; (2) the right P5 basis has hyaline lamella on the inner margin but without chitinous prominence; (3) the inner distal margin of the P5 intercoxal sclerite is produced into a protruded plate; and (4) the right caudal ramus has ventral chitinous processes.
2. The *gladiolus* species group includes *M.calcarus*, *M.dumonti*, *M.gladiolus*, and *M.rarus* and exhibits the following characteristics: (1) the spinous process on the antepenultimate segment of the right antennule is comb-like; (2) the right P5 basis has no inner hyaline membrane; (3) the inner distal margin of the P5 intercoxal plate is not produced into a protruded plate; and (4) the right P5 Exp-1 has no acute process on the outer distal margin.
3. The *mephistopheles* species group includes *M.mephistopheles*, *M.uenoi*, and *M.pectinidactylus* and exhibits the following characteristics: (1) the spinous process on the antepenultimate segment of the right antennule is comb-like; (2) the male right P5 Exp-2 has a straight or bent principal lateral spine; and (3) both the right and left basis have an inner hyaline membrane.
4. The *loeiensis* species group includes *M.loeiensis*, *M.mekongensis*, and *M.phutakaensis* sp. nov. and exhibits the following characteristics: (1) the spinous process on the antepenultimate segment of the right antennule is comb-like; (2) the male right P5 Exp-2 has enlarged proximal and distal parts of the outer margin and a bent and twisted principal lateral spine; (3) the right P5 coxa has a strong spine; (4) the left P5 basis has an inner hyaline lamella; and (5) the right P5 Exp-1 has an acute process on the outer distal margin.

### ﻿Biogeography

Regarding distribution records of *Mongolodiaptomus* species, *M.phutakaensis* sp. nov. is the 13^th^ member of the genus and the 10^th^ taxon recorded in Thailand. Only three species (*M.birulai*, *M.gladiolus*, and *M.mephistopheles*) among the 13 species recorded across Asia remain unrecorded in Thailand (Sanoamuang and Dabseepai 2021). Previous records of *M.mephistopheles* by Bricker et al. (1978) and Boonsom (1984) from Thailand were actually misidentified specimens of *M.calcarus* (Ranga Reddy et al. 2000). Thailand is the most species-rich country with *Mongolodiaptomus* in Southeast Asia. From Vietnam are known seven valid species (Boonmak and Sanoamuang 2022), plus two unnamed species (Tran et al. 2016). China has six species (Li et al. 2018), including a newly recorded taxon, *M.mekongensis*, from Hainan Island by Wei et al. (2023). Cambodia, and Malaysia have five species each (Sanoamuang and Watiroyram 2018; Chaicharoen and Sanoamuang 2022). Laos has four species, while Indonesia and Taiwan have three species each, whereas Singapore and the Philippines both have only one species each (Lopez et al. 2017; Sanoamuang and Watiroyram 2018).

Most species are currently restricted to Southeast Asia; only six species have also been recorded outside the area, including *M.birulai*, *M.calcarus*, *M.gladiolus*, *M.mekongensis*, *M.pectinidactylus*, and *M.uenoi* (Ranga Reddy et al. 1998, 2000; Sanoamuang 2001; Alekseev et al. 2013; Tran et al. 2016; Lopez et al. 2017). Therefore, the distribution of these six species extends from Southeast Asia to South China. *M.birulai* has the widest distribution, occurring from Vietnam upwards to North China, including Taiwan (Young and Shih 2011; Tran et al. 2016; Li et al. 2018). *M.mekongensis* is a common species in the Mekong region and has been found in Thailand, Laos, Cambodia, Vietnam, and South China (Table 4). *M.botulifer* and *M.malaindosinensis* have been found only in Southeast Asia (Boonmak et al. 2018). *M.dumonti*, and *M.mephistopheles*, are confined in distribution to the Mekong region, and Malay Archipelago, respectively. Three species (*M.loeiensis*, and *M.phutakaensis* sp. nov., and *M.rarus*) are currently endemic to Thailand; for more details see also Table 2 in Sanoamuang and Watiroyram (2018).

In Thailand, the most widespread *Mongolodiaptomus* species are *M.botulifer* and *M.calcarus*. Both species live in both temporary and permanent water bodies throughout the country and occur throughout the year. *M.malaindosinensis* is moderately common and has been recorded throughout the country. *M.mekongensis* is widely distributed in the Mun River Basin, a tributary of the Mekong River, and mostly occurs in temporary water bodies. *M.dumonti*, *M.rarus*, and *M.uenoi*, are uncommon. *M.rarus* has been found only in temporary water bodies. In contrast, *M.pectinidactylus* is rare and has been reported only at two temporary water bodies (Sanoamuang 2002). *M.loeiensis* and *M.phutakaensis* sp. nov. are extremely rare and, to date, have been found only in a single locality each (Watiroyram and Sanoamuang 2017; this study).

## Supplementary Material

XML Treatment for Mongolodiaptomus phutakaensis

## ﻿Additional information

### Author ORCIDs

Laorsri Sanoamuang https://orcid.org/0000-0003-0377-1225

Kamonwan Koompoot https://orcid.org/0009-0005-2116-5191

### Data availability

All of the data that support the findings of this study are available in the main text.
